# *Eucalyptus globulus* and *Salvia officinalis* Extracts Mediated Green Synthesis of Silver Nanoparticles and Their Application as an Antioxidant and Antimicrobial Agent

**DOI:** 10.3390/plants11081085

**Published:** 2022-04-15

**Authors:** Aistė Balčiūnaitienė, Mindaugas Liaudanskas, Viktorija Puzerytė, Jonas Viškelis, Valdimaras Janulis, Pranas Viškelis, Egidijus Griškonis, Virginija Jankauskaitė

**Affiliations:** 1 Lithuanian Research Centre for Agriculture and Forestry, Institute of Horticulture, 54333 Babtai, Lithuania; viktorija.puzeryte@lammc.lt (V.P.); jonas.viskelis@lammc.lt (J.V.); pranas.viskelis@lammc.lt (P.V.); 2 Department of Pharmacognosy, Faculty of Pharmacy, Lithuanian University of Health Science, 44307 Kaunas, Lithuania; mindaugas.liaudanskas@lsmuni.lt (M.L.); valdimaras.janulis@lsmuni.lt (V.J.); 3 Institute of Pharmaceutical Technologies, Faculty of Pharmacy, Lithuanian University of Health Science, 50166 Kaunas, Lithuania; 4 Department of Physical and Inorganic Chemistry, Kaunas University of Technology, 50254 Kaunas, Lithuania; egidijus.griskonis@ktu.lt; 5 Department of Production Engineering, Kaunas University of Technology, 51424 Kaunas, Lithuania; virginija.jankauskaite@ktu.lt

**Keywords:** green synthesis, *Eucalyptus globulus*, *Salvia officinalis*, phytochemical analysis, silver nanoparticles, antioxidant activity, antibacterial activity

## Abstract

Silver nanoparticles (AgNPs) biosynthesized using plant extracts as reducing and capping agents show multiple possibilities for solving various biological problems. The aim of this study was to expand the boundaries of AgNPs using a novel low toxicity and production cost phytochemical method for the biosynthesis of nanoparticles from *Eucalyptus globulus* and *Salvia officinalis* aqueous leaf extracts. Biosynthesized AgNPs were characterized by various methods (ultraviolet-visible spectroscopy (UV-vis), Fourier transform infrared (FTIR) spectroscopy with horizontal attenuated total reflectance (HART), transmission electron microscopy (TEM), energy-dispersive X-ray spectroscopy (EDS)). The determined antioxidative and antimicrobial activity of plant extracts was compared with the activity of the AgNPs. The UV-vis spectral analysis demonstrated the absorption peaks at 408 and 438 nm, which confirmed the synthesis of stable AgNPs from *E. globulus* and *S. officinalis,* respectively. FTIR-HART results suggested strong capping of phytochemicals on AgNPs. TEM results show mainly spherical-shaped AgNPs, whose size distribution depends on the plant leaf extract type; the smaller AgNPs were obtained with *E. globulus* extract (with size range of 17.5 ± 5.89 nm compared to 34.3 ± 7.76 nm from *S. officinalis* AgNPs). The in vitro antioxidant activity evaluated by radical scavenging assays and the reduction activity method clearly demonstrated that both the plant extracts and AgNPs showed prominent antioxidant properties. In addition, AgNPs show much stronger antimicrobial activity against broad spectrum of Gram-negative and Gram-positive bacteria strains than the plant extracts used for their synthesis.

## 1. Introduction

In recent years, there has been an increasing focus on biomedicine and other sciences by studying the chemical diversity of plants and the raw materials they provide. The results of the research are important in the search for plant raw materials, in the development of medicinal products and food supplements of plant origin, and in finding new possibilities for the use plants and plant raw materials [1,2].

Green synthesis represents an environmentally benign, low production cost, relatively reproducible, simple method [3,4,5,6]. For obtaining plant-derived nanomaterials, the plant biodiversity is broadly regarded due to the selection of “green reagents” such as ketones, aldehydes, amides, phenols, flavonoids and terpenoids, carboxylic acids, and ascorbic acids, which are available in a huge class of plant extracts, particularly in plant leaves [2,7,8,9,10]. Eucalyptus and sage leaves are very valuable raw plant materials that possess many biological activities and are promising agents for the green synthesis of AgNPs [11].

It has been indicated in the scientific literature that extracts of sage have particularly strong antioxidant activity compared to other herbal plants [12]. This effect is primarily related to the complex of different groups of phenolic compounds (phenolic acids, luteolin, apigenin, quercetin, isorhamnetin glycosides) accumulated in sage leaves [13]. These extracts also show a strong anti-inflammatory effect due to the triterpene compounds accumulated in raw material, especially ursolic acid. Many studies have shown that extracts of sage have biological activity against various bacteria strains and yeasts [13]. Sage leaf extracts also have antispasmodic, hypoglycemic, and antimutagenic effects. Sage tea is used as a traditional herbal medicine to relieve the symptoms of inflammation of the mucous membranes of the mouth and throat, and to reduce heavy hyperhidrosis, dizziness, and mild symptoms of indigestion; recent studies indicate wider benefits [14,15,16,17].

Eucalyptus is one of the most widely utilized medicinal plants due to its wide spectrum of biological activities, which are mainly attributed to the diversity of phytochemical constituents in the plant parts. Eucalyptus leaf decoctions are used for the mouth cavity, rinsing wounds, compresses in trauma, upper respiratory tract pain, and rheumatism; it is suitable for inhalations in upper respiratory tract diseases, bronchitis. The biologically active compound complex found in eucalyptus leaves determines a particularly strong antioxidant effect of their extracts [18,19,20]. The most important phenolic compounds that largely determine the antiradical and reductive activity of eucalyptus leaves are phenolic acids, quercetin glycosides [21], and other groups of phenolic compounds of various structures [22]. Like sage leaf extract, eucalyptus leaf extract has extremely strong antibacterial and antifungal activity [23].

In general, the chemical, physical, mechanical, and antimicrobial properties of AgNPs depend on their chosen precursor and synthesis method [24]. Synthesis of nanoparticles can be achieved by many different methods, such as reduction in solutions (distilled water, ethanol, hexane, toluene, ethylene glycol, and others), electrochemical and sonochemical approaches [25,26], the microwave-assisted method (by choosing the right power, time, and medium) [27], the radiation-assisted method [8], and via biological routes and chemical reactions [28]. In general, nanoparticles are prepared by a variety of chemical and physical methods, but these approaches are expensive and harmful to the environment due to their use of perilous and toxic chemicals, which are responsible for biological risks and hazardous to human health [29]. However, AgNPs are mainly used in healthcare and medicine due to their strong antibacterial (inhibits DNA replication, enzyme functions, etc.), antiviral (blocks the attachment of viruses to the cell wall), antifungal (destroys the cell membrane), and other activities [30,31]. Therefore, it is imperative to develop non-hazardous, economical, and feasible methods for the synthesis of AgNPs to maximize their potential benefits to society.

The present paper reports the biological synthesis of AgNPs using the aqueous leaf extracts of *Eucalyptus globulus* Labill. (*E. globulus*) and *Salvia* *officinalis* L. (*S. officinalis*), and evaluation of the nanoparticles’ potential application as an antibacterial agent against various pathogenic bacteria strains, having an antioxidant potential.

## 2. Results and Discussion

### 2.1. Phytochemical Analysis and Morphology of Leaf Extracts

Silver nanoparticles were successfully synthesized by the reduction of AgNO_3_ with the leaf extracts of *E. globulus* (*EuG*-AgNPs) and *S. officinalis* (*SaO*-AgNPs). Various bioactive derivatives presented in leaf extracts are responsible for the bioreduction and capping/stabilization of AgNPs [32,33]. Among the most important biologically active compounds accumulated by plants are phenolic compounds, which are classified as natural antioxidants [34,35]. Oxidative stress causes changes in cellular metabolism associated with DNA and protein damage and lipid peroxidation [36,37]. Phenolic compounds neutralize reactive forms of oxygen and nitrogen [38,39], and have antimicrobial [40,41] anti-inflammatory effects [42,43]; they are therefore valuable in the prevention and treatment of many diseases. For this reason, studies on the qualitative and quantitative composition of plant raw materials that accumulate phenolic compounds are important and relevant.

To evaluate phytochemical composition, the UV-VIS spectrophotometric evaluation of the total composition of phenolic compounds, flavonoids, proanthocyanidins, and hydroxycinnamic acid derivatives in *E. globulus* and *S. officinalis* extracts, and AgNPs from these extracts, was performed. The obtained results are summarized in Table 1. The highest content of phenolic compounds was found in *S. officinalis* leaf extract (0.78 mg GAE/g), whereas a slightly lower amount (in 12%) was detected in the *E. globulus* extract. The results of phytochemical composition obtained for *E. globulus* and *S. officinalis* frequently differ from those obtained by other researchers [44,45]. This can be explained by the fact that other factors not studied may strongly affect the qualitative and quantitative content of the vegetative and generative organs of various plants; for example, the geochemical composition of the soil, the geographic region, the climate and meteorology, plant cultivation and raw material storage conditions, and foliar fertilization lead to increased phenol content. 

A similar dependence is characteristic for the content of hydroxycinnamic acid derivatives in *S. officinalis* and *E. globulus* extracts (1.57 and 1.38 mg CAE/g, respectively). Furthermore, *E. globulus* extract contains a larger amount of proanthocyanidin and flavonoid compounds compared to *S. officinalis* extract.

The total content of phenol, flavonoid, hydroxycinnamic acid, and proanthocyanidins after biosynthesis of *EuG*-AgNPs and *SaO*-AgNPs is lower compared to that of pure *E. globulus* and *S. officinalis* leaf extracts, but the character of changes is the same (Table 1). A higher content of total hydroxycinnamic acid and phenolic compounds was found in *SaO*-AgNPs, whereas slightly higher contents of proanthocyanidins and flavonoids were detected in the *EuG*-AgNPs. The content of phenolic compounds in the case of nanoparticles is 15–20% lower than that of plant extracts. It is supposed that the donating potential of polyphenols facilitates the formation of nanoparticles by bioreduction of Ag^+^ to Ag^0^ and further stabilization of nanoparticles [46].

**Figure 1:**
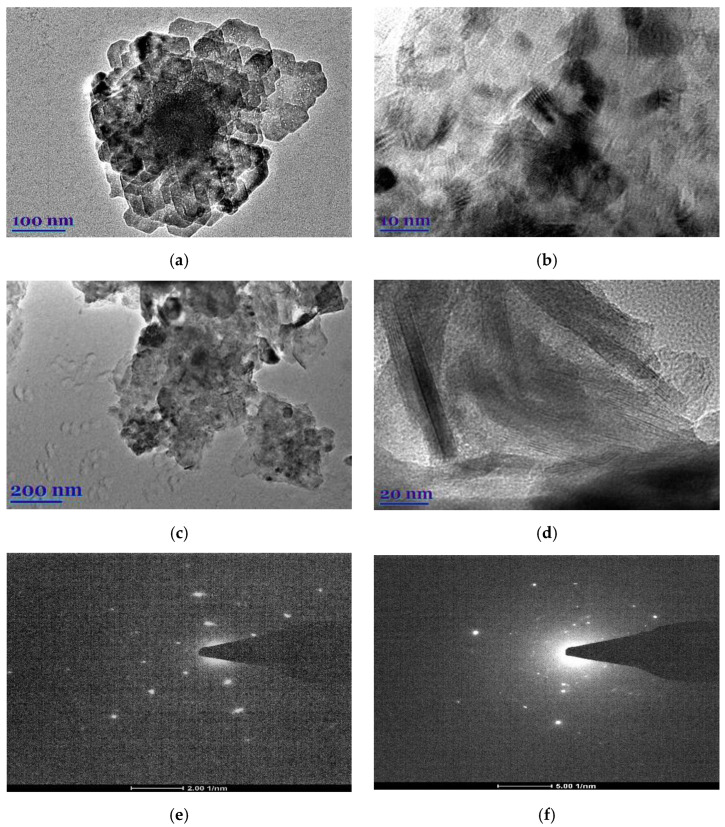
TEM images (**a**–**d**) and SAED (**e**,**f**) of *E. globulus* (**a**,**b**,**e**) and *S. officinalis* (**c**,**d**,**f**) patterns.

The morphological peculiarities of leaf extracts were examined by TEM and selected area electron diffraction (SAED). Figure 1a–d shows TEM images of *E. globulus* and *S. officinalis* leaf extracts. It was found that the natural compounds from extracts show highly ordered structures. In the case of *E. globulus*, nano-sized octagonal-shaped crystallites of 40 to 55 nm in size are closely aligned. In the case of *S. officinalis*, the needle-shaped crystallites are visible, whose longitudinal dimensions are considerably larger than those of the *E. globulus* derivatives. Thus, the obtained *E. globulus* and *S. officinalis* extracts are highly crystalline, as shown by clear lattice fringes and typical SAED (Figure 1e,f).

### 2.2. Morphological Analysis of Biosynthesized AgNPs

**Figure 2:**
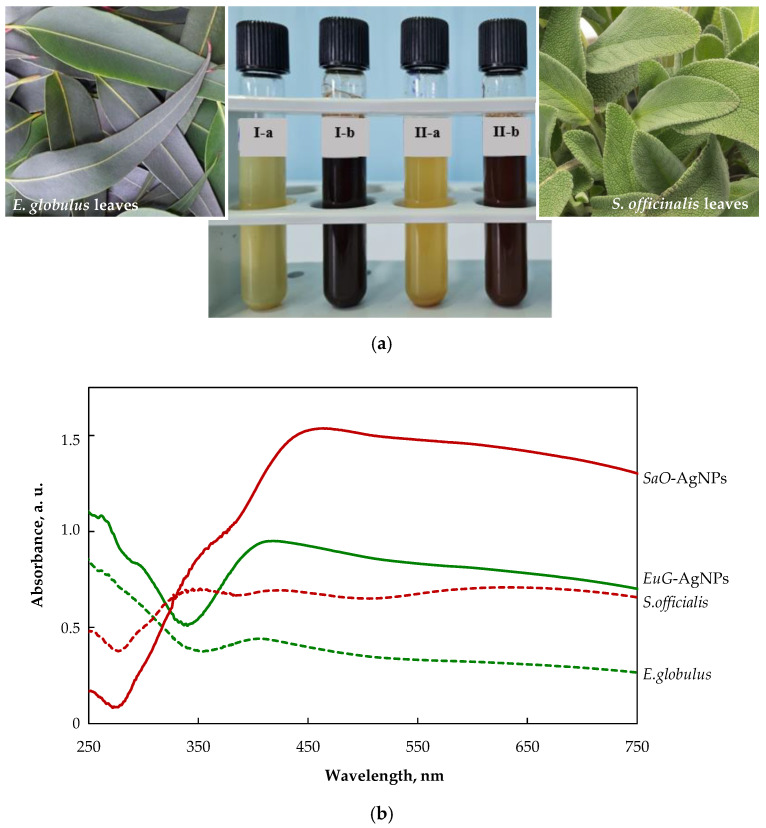
(**a**) Changes in the color of the colloidal solutions indicating the formation of AgNPs (I-a—*E. globulus* + AgNO_3_; I-b—*EuG*-AgNPs; II-a—*S. officinalis* +AgNO_3_; II-b—*SaO*-AgNPs); (**b**) UV-vis absorption spectra of plant extracts and biosynthesized AgNPs.

The color change of colloidal solutions from yellowish to reddish brown and dark brown visually evidences the formation of *SaO*-AgNPs and *EuG*-Ag NPs, respectively (Figure 2a). The color change of nanoparticles’ colloidal solutions happens due to localized surface plasmon resonance (LSPR) after the bioreduction of Ag^+^ ions to Ag^0^ by phytochemicals. It was confirmed by UV-visible spectroscopic analysis, as shown in Figure 2b. The biosynthesized *SaO*-AgNPs that were stabilized with *S. officinalis* extract show a strong LSPR peak at 438 nm, whereas for *E. globulus* extract, the stabilized *EuG*-AgNPs peak appears at 408 nm. Generally, the intensity and position of LSPR are dependent on the shape and size of nanoparticles, and the composition of the surrounding medium [47]. It is proposed that smaller nanoparticles primarily absorb light and have peaks near 400 nm, whereas larger spherical particles exhibit increased scattering and have peaks that broaden and shift towards longer wavelengths [48]. Thus, it is suggested that *EuG*-AgNPs should be smaller in size than *SaO*-AgNPs.

**Figure 3:**
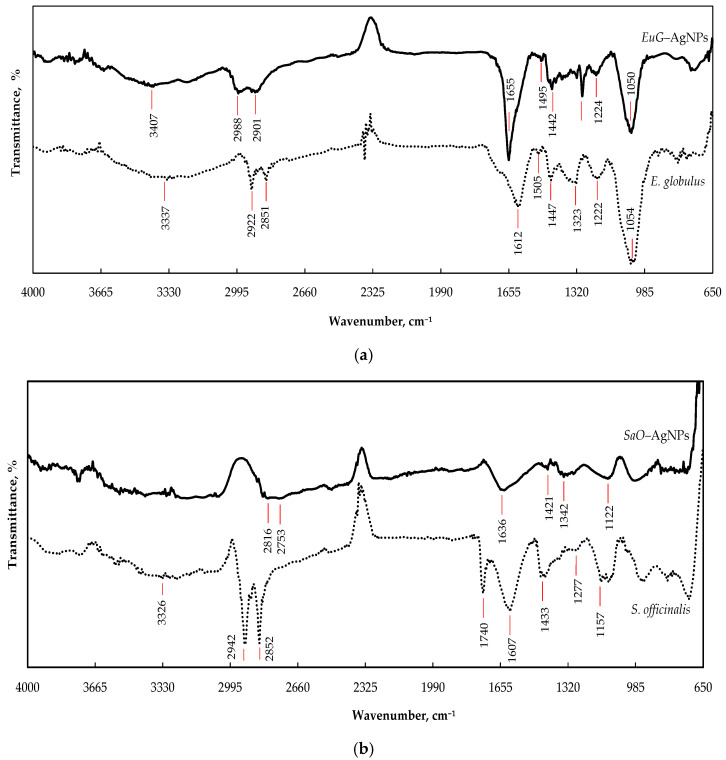
FTIR spectra of AgNPs synthesized by the reduction of AgNO_3_ with the *E. globulus* (**a**) and *S. officinalis* (**b**) leaf extracts.

FTIR-HART analysis was used to identify the role of functional groups presented in *E. globulus* and *S. officinalis* extracts in the bioreduction, capping formation, and stabilization of AgNPs. Figure 3a demonstrates the possible functional groups of *E. globulus* extract. The wide absorbance band with a peak at 3337 cm^−1^ is attributed to the O–H stretching vibration of alkynes, alcohols, and phenols [49,50], whereas the overlapping peaks at 2922 and 2851 cm^−1^ can be assigned to the C–H stretching vibrations in CH/CH_2_/CH_3_ groups [51]. The absorbance bands in the region of 1600–1490 cm^−1^ correspond to C=C stretching vibrations of aromatic rings [51,52]. The peak observed at 1612 cm^−1^ can be attributed to N–H bending vibrations of alkenes, primary amines, and amides, and the peak at 1505 cm^−1^ can be assigned to N=O stretching of nitro groups (NO_2_) and N–H bending of secondary amines [49,51]. The peak at 1447 cm^−1^ is related to the C–H bending vibration of proteins. The peak at 1323 cm^−1^ may occur due to the bending vibration of nitro groups (N=O); the peak at 1222 cm^−1^ indicates the presence of the stretching vibration of the C–O bond from alcohols, esters, carboxylic acid, or ether [49,50]; and the prominent peak at 1054 cm^−1^ is typical for the C–N (amines) stretching vibration of proteins [52]. During formation of *EuG*-AgNPs, biomolecules of phytochemicals bind with the silver through various functional groups and form a coating on the surface of the nanoparticles. Therefore, in the FTIR spectrum of biosynthesized *EuG*-AgNPs, similar peaks as those of the spectrum of *E. globulus* leaf extract appear (Figure 3a). *EuG*-AgNPs’ spectrum shows the presence of O–H, C–H, C=O, N–H, N=O, C–O, and C–N functional groups, with peaks at 3407, 2988, 2901, 1655, 1495, 1442, 1293, 1224, and 1050 cm^−1^, respectively.

The spectral bands that reflect the chemical composition of *S. officinalis* extract can be seen from the FTIR spectrum shown in Figure 3b. The bands between 3000 and 3600 cm^−1^ are mainly due to OH stretching vibrations within phenols [53]. Two high intensity bands at 2924 and 2850 cm^−1^ can be attributed to methylene −CH_2_– and methyl −CH_3_ groups, respectively, whereas the carbonyl of the triglyceride linkage ester is observed at 1740 cm^−1^ [54]. The absorption peak appearing at 1607 cm^−1^ may be due to C=O bond stretching within the aromatic rings of different phenolic compounds present in *S. officinalis*. The region between 1500 and 1200 cm^−1^ includes mixed vibrations arising from the bending modes of the >CH_2_ and −CH_3_ groups in proteins, fatty acids, and phosphate-bearing compounds. The presence of carbohydrates and polysaccharides reveals the vibrations of the −C–O–C glycoside ring bond, C–O stretching in COOH, and O–H bending in the region from 1220 to 1000 cm^−1^ [54,55]. The spectral range between 900 and 650 cm^−1^ corresponds to the vibrations of bands arising from amino acids and nucleotides [54]. The FTIR spectrum of the *SaO*-AgNPs synthesized shows peaks at 3312, 2824, 2753, 1636, 1421, 1339, and 1121 cm^−1^, which are identical to the peaks that appear in the spectrum of *S. officinalis* leaf extract and indicate the presence of the organic compounds of the phytochemicals (Figure 3b).

Some changes in the FTIR spectra of the biosynthesized *EuG*-AgNPs and *SaO*-AgNPs, such as the decrease in the absorption bands’ intensity, and their shifting or disappearing comparing to the absorption bands of the pure plant extracts, confirm the involvement of biomolecules of phytochemicals in the bioreduction, capping, and stabilization of AgNPs. The organic compounds such as flavanones or terpenoids may be responsible for the bioreduction of nanoparticles, whereas proteins can be involved in the stabilization of AgNPs [49]. It is believed that proteins can bind to AgNPs through free amine groups or cysteine residues in the proteins [56], whereas flavanones and terpenoids are absorbed on the surface of AgNPs, possibly by interaction through carbonyl [51,57,58]. It was determined that terpenoids reduce metal ions by oxidation of aldehydic groups in the molecules to carboxylic acid [57].

**Figure 4:**
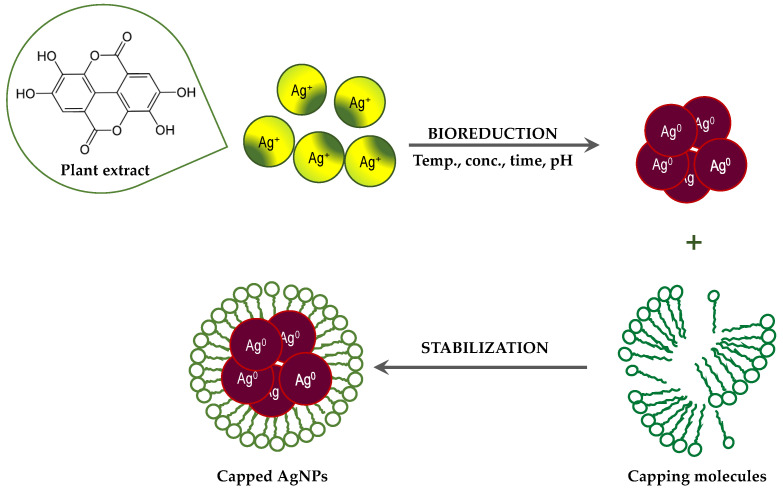
Suggested mechanisms for the formation of AgNPs via bioreduction derived from plant extracts.

Although the process of biosynthesis of nanoparticles remains unclear, plausible mechanisms have been proposed to explain the formation of metallic AgNPs via bioreduction derived from plants [32,33]. Plants are rich in secondary metabolites, such as flavonoids, tannins, and phenolic acid. Many of these metabolites act as both reducing and stabilizing agents, and inhibit the aggregation of formed nanoparticles [32,59]. Generally, the Ag^+^ ions are inactivated via phytochelation, probably dye to the nucleophilic nature of the phenolic aromatic rings. It is supposed that ions are captured and immobilized by biological elements and subsequently undergo reduction, growth, and sintering processes, leading to the formation of AgNPs. Thus, the bioreduction of AgNPs by plant extracts can be divided into three phases [32]: (a) the activation phase, in which reduction and nucleation of Ag^+^ ions occurs; (b) the growth phase, when the small neighboring AgNPs combine to form larger particles, accompanied by an increase in the thermodynamic stability of the AgNPs; and (c) the termination phase, in which the final shape of the AgNPs is formed through (Figure 4).

**Figure 5:**
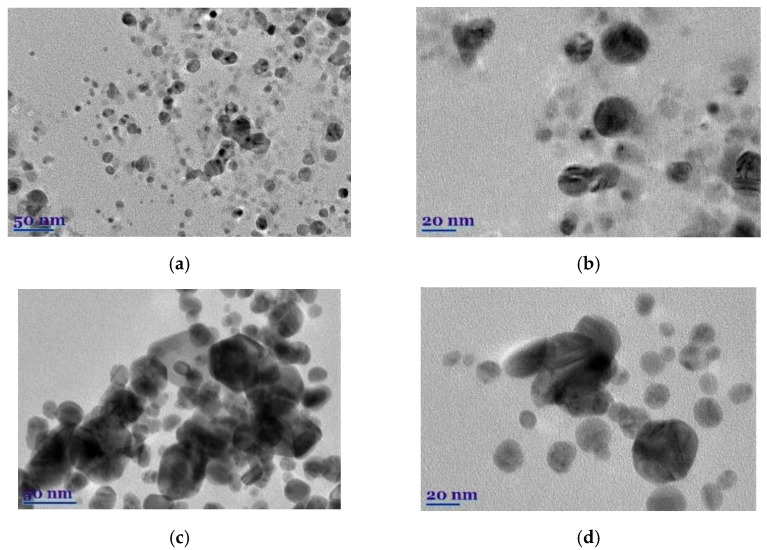

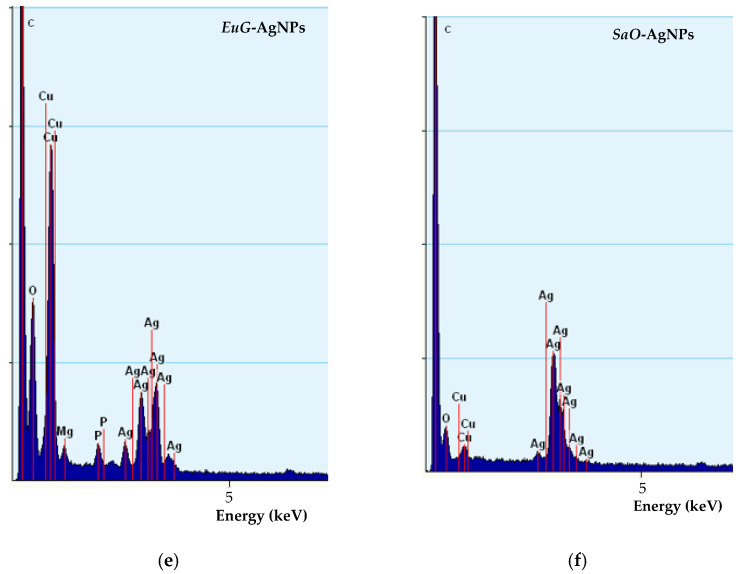
TEM images (**a**–**d**) and EDS spectra (**e**,**f**) of biosynthesized *EuG-*AgNPs (**a**,**b**,**e**) and *SaO*-AgNPs (**c**,**d**,**f**).

The morphology, shape, size, and chemical composition of biosynthesized *EuG*-AgNPs and *SaO*-AgNPs were examined by TEM and TEM-EDS. It is postulated that the type of plant extract and its concentration influence the morphology of the nanoparticles formed, whereas the temperature and pH of the extract medium controls the growth and size of the nanoparticles [32]. The shape, size, and morphology of green synthesized *EuG*-AgNPs and *SaO*-AgNPs are shown in Figure 5a–d. This shows that most of the nanoparticles are nearly spherical, whereas some of *EuG*-AgNPs and *SaO*-AgNPs have a triangular or hexagonal shape. A slight agglomeration of nanoparticles is also observed. After evaluating the particle distribution using the ImageJ application, it was found that the size of the nanoparticles depends on the plant extract used for bioreduction and stabilization. The diameter of the *EuG*-AgNPs was found to be in the range of 17.5 ± 5.89 nm (polydispersity approx. 4.5), whereas the *SaO*-AgNPs diameter is almost twice large and is in the range of 34.3 ± 7.76 nm (polydispersity approx. 6.0). The obtained results prove the suggestion that larger *SaO*-AgNPs show a UV-vis absorption peak at a longer wavelength (see Figure 2b).

TEM-EDS spectra confirm the presence of biosynthesized nanoparticles (Figure 5e,f). *EuG*-AgNPs and *SaO*-AgNPs show a strong peak at approximately 3 keV, which is characteristic of metal silver due to LSPR [60]. In addition, other peaks for oxygen, carbon, and copper (from the TEM grid) were also observed.

### 2.3. Comparison of Bioactivity of the Plant Extracs and Biosynthesized AgNPs

#### 2.3.1. Antioxidant Activity

Plant extracts have a variety of compounds that act as antioxidants through different reaction mechanisms. Therefore, antioxidant activity cannot be adequately tested using only one method. In the scientific literature, it is strongly recommended to use at least two different methods for the determination of antioxidant activity in plant extracts [61]. Therefore, in this study, four different antiradical and reduction activity determination methods were used for an in-depth evaluation of the antioxidant activity of the analyzed *E. globulus* and *S. officinalis* extracts. ABTS, DPPH, and TFPH assays are based on the ability of antioxidants to scavenge ABTS^•+^, DPPH^•^, and TFPH^•+^ free radicals [62,63], whereas the FRAP assay measures the reduction activity of antioxidants [64]. The ABTS^•+^ radical cation scavenging assay enables the determination of the antiradical activity of both lipophilic and hydrophilic antioxidants at a medium pH of 7.4 (medium pH is close to blood pH of 7.35–7.45) [63]. Furthermore, the DPPH^•^ radical scavenging method is only suitable for evaluating the in vitro antiradical activity of compounds soluble in organic solvents. However, it limits the evaluation of antioxidant activity of hydrophilic antioxidants [65,66]. The TFPH^•+^ method is used to evaluate the activity of biologically active compounds in a strongly acidic medium [67].

The antioxidant activity of eucalyptus and salvia species has been reported by many researchers [17,18]. The antioxidant capacity of the studied *E. globulus* and *S. officinalis* leaf extracts is shown in Table 2. The *E. globulus* extract showed stronger in vitro antioxidant activity compared to the *S. officinalis* extract. This tendency is especially pronounced after the FRAP assay, when *E. globulus* showed more than 2 times stronger reducing activity than *S. officinalis* (9.23 and 4.23 mmol TE/g, respectively). The *E. globulus* extract also showed stronger antiradical activity evaluated by ABTS^•+^, DPPH^•^, and TFPH^•+^ radical scavenging methods. The antioxidant activity observed with the extracts of *E. globulus* and *S. officinalis* leaves is probably due to phenolic and proanthocyanidins compounds, which are well known for their antioxidant capacity [68,69].

Further, the antioxidant activity of biosynthesized AgNPs was compared to the antioxidant activity of *E. globulus* and *S. officinalis* leaf extracts (Table 2). Data obtained by ABTS, DPPH, and TFPH in vitro assays show that biosynthesized AgNPs possess higher antioxidant potential due to the existence of silver in two oxidation states, Ag^+^ and Ag^2+^, and phytochemicals capped on the AgNPs’ surface [46]. *EuG*-AgNPs show stronger antioxidant capacity than *SaO*-AgNPs, with the exception of the antiradical activity determined by the ABTS method. According to the FRAP assay result, it is clear that both AgNPs indicate slightly lower reducing activity than the plant extracts.

#### 2.3.2. Antibacterial Activity

It was determined that eucalyptus [70,71] and salvia [72,73] species exhibit antibacterial activity against various pathogens, and are promising alternatives to the use of hazardous chemicals, and may have potential applications in food, pharmaceutical products, etc. In this study, the antibacterial activity of *E. globulus* and *S. officinalis* leaf extracts and biosynthesized AgNPs was investigated against both Gram-positive (*S. aureus*, *B. cereus,* *P. vulgaris*, *B. subtilis*) and Gram-negative (*E. coli*, *P. aeruginosa*, *K. pneumoniae*, *P. mirabilis*) bacteria strains (Table 3). The results revealed that *E. globulus* and *S. officinalis* extracts are potentially effective in suppressing bacterial growth with a range of inhibition zone from 12.5 to 17.9 mm. Accordingly, *E. globulus* and *S. officinalis* extracts were found to show slightly higher antibacterial activity against Gram-positive bacteria than Gram-negative bacteria (*p*  >  0.05) due to the variation in their cell wall structure [74,75]. *E. globulus* extract exhibits a stronger inhibitory effect against most pathogens, except *S. aureus* and *B. cereus*. Importantly, a high inhibition ability of *E. globulus* and *S. officinalis* was detected against *K. pneumoniae* and *P. aeruginosa*, which are biofilms that cause organisms’ resistance to multiple classes of antibiotics and can cause nosocomial infections [76]. Various mechanisms have been suggested to explain the mode of action of plant antimicrobials. Generally, these include damaging the bacterial cell membrane, inhibiting efflux pumps, and inhibiting DNA and protein biosynthesis [33].

As can be seen from Table 3, biosynthesized *EuG*-AgNPs and *SaO-*AgNPs demonstrate 25–45% higher antibacterial activity against all tested pathogenic microorganisms compared to that of pure *E. globulus* and *S. officinalis* extracts. Comparing antibacterial properties between nanoparticles, *SaO*-AgNPs more effectively prevent the growth of bacteria tested, with a range of inhibition zone from 18.8 to 24.4 mm, whereas for *EuG*-AgNPs, the inhibition zone varies from 18.2 to 21.5 mm. It can be assumed that a higher content of hydroxycinnamic acid and phenolic compounds in the *S. officinalis* extract increases the antibacterial capacity of *SaO*-AgNPs. In addition, the antimicrobial activity of AgNPs also depends on the nanoparticle morphological characteristics, such as the shape and size [77,78].

The antibacterial mode of action of silver nanoparticles against bacteria is also not clearly understood. There are several hypotheses for the nanoparticles’ effect on the cell, such as their adhesion on the surface of the bacterial cell wall and membrane, penetration into the cell and disruption of intracellular organelles and biomolecules, induction of oxidative stress, and modulation of signal transduction [19,20,22,28,79].

Thus, AgNPs obtained by the reduction of AgNO_3_ with *E. globulus* and *S. officinalis* extracts have a broad spectrum of activity by inhibiting Gram-positive and Gram-negative bacteria strains.

## 3. Materials and Methods

### 3.1. Chemicals

Silver nitrate (AgNO_3_), ABTS^•+^ (2,2′-azino-bis(3-ethylbenzothiazoline-6-sulphonic acid)), sodium acetate trihydrate (CH_3_COONa × 3H_2_O), iron(III) chloride hexahydrate (FeCl_3_ × 6H_2_O), TPTZ (2,4,6-Tris(2-pyridyl)-*s*-triazine), TFPH (trifluoperazine hydrochloride), and Trolox (6-hydroxy-2,5,7,8-tetramethylchroman-2-carboxylic acid were purchased from Merck (Darmstadt, Germany); ethanol (96.3% *v*/*v*) was obtained from Stumbras, AB (Kaunas, Lithuania); potassium chloride (KCl) was obtained from Scharlau (Barcelona, Spain); potassium bisulfate (K_2_S_2_O_8_) and DPPH^•^ (2,2-Diphenyl-1-(2,4,6-trinitrophenyl)hydrazin-1-yl) were obtained from Alfa Aesar GmbH & Co KG (Karlsruhe, Germany); sulfuric acid (H_2_SO_4_, 95% was purchased from Chempur (Piekary Śląskie, Poland). All chemicals used were of analytical grade.

### 3.2. Plant Materials

Finely cut *Salvia officinalis* (Švenčionių vaistažolės UAB, Švenčionys, Lithuania) and *Eucalyptus globulus* (Acorus Calamus UAB, Pakruojis, Lithuania) leaves were purchased from a public pharmacy operating in Kaunas (Lithuania). Plant material was ground to a powder using a mill (IKA^®^ A11 basic, Staufen, Germany). Loss on drying before analysis was determined by drying about 1 g of powdered raw plant material in a moisture analyzer (Precisa HA 300, Precisa Instruments AG, Dietikon, Switzerland) to complete evaporation of water and volatile compounds at a drying temperature of 105 °C. The data were recalculated for absolute dry weight (DW).

### 3.3. Preparation of Plant Leaf Extracts

A quantity of 125 g of raw material (crushed *E. globulus* or *S. officinalis* leaves) was soaked for 3 h in 70% (*v*/*v*) ethanol. The soaked raw material was transferred to a percolator, covered with extractant, i.e., 70% (*v*/*v*) ethanol, and left to macerate for 48 h. Then, it was percolated at speed of 0.3 mL/min and high-concentration extract (85% of total extract amount) was obtained. Low-concentration extract was decanted until all biologically active substances were washed from the raw material. It was evaporated using an IKA^®^ HB 10 rotary evaporator (IKA^®^-Werke GmbH & Co. KG, Breisgau, Germany) up to 15% of the total liquid extract amount. The remaining part of the low-concentration extract was transferred to a single container with the high-concentration extract, and liquid extract for antioxidant activity analysis was obtained. Ethanolic phase of *E. globulus* and *S. officinalis* leaf liquid extracts was evaporated at 70 °C for 4 h by a rotary evaporator (Buchi Rotavapor R-205, Buchi AG, Flawil, Switzerland) and aqueous phase extracts of yellowish color were obtained. These aqueous extracts were used as green reductants and capping agents for the biosynthesis of AgNPs. In addition, a portion of *E. globulus* and *S. officinalis* aqueous extracts was lyophilized at 0.01 mbar pressure and condenser temperature of −85 °C using a Zirbus lyophilizer (Zirbus technology GmbH, Bad Grund, Germany) for morphological studies.

### 3.4. Green Synthesis of Silver Nanoparticles

A quantity of 0.03 g of AgNO_3_ was dissolved in 2.5 mL distilled water and mixed with 30 mL of *E. globulus* or *S. officinalis* aqueous extract under vigorous stirring at room temperature and speed of 400 rpm for 2 h. The mixtures were incubated at room temperature for 24 h, and the change in color from yellowish to brown proved the formation of *EuG*-Ag NPs and *SaO*-AgNPs. After that, the material was stored in darkness at a 6 °C temperature.

### 3.5. Characterization of Leaf Extracts and AgNPs

The formation of *EuG*-AgNPs and *SaO*-AgNPs was checked using a Lambda 25 UV-vis spectrometer (PerkinElmer, Waltham, MA, USA). The analysis was performed in the wavelength range from 200 to 800 nm.

The functional characterization of materials to be tested was performed by Fourier transform infrared (FTIR) spectrometry. Spectra were recorded using the Spectrum GX FTIR spectrometer (PerkinElmer, Waltham, MA, USA), which was equipped with a horizontal attenuated total reflection (HATR) accessory. The FTIR-HATR spectra of samples were recorded at room temperature in the wave number range of 4000–600 cm^−1^ with a resolution of 1 cm^−1^. Collected spectra were processed with the Spectrum^®^ v5.0.1 software from the PerkinElmer.

The microscopy analysis was applied for understanding morphological and structural features of the tested materials. A Tecnai G2 F20 X-TWIN transmission electron microscope (TEM) (FEI, Hillsboro, OR, USA) was used to examine the size and shape of the synthesized AgNPs. The diluted sample was deposited drop-wise onto carbon-coated copper TEM grids. The Schottky emission electron source was used with the accelerating voltage of 20–200 kV. The resolution of the microscope ranged from 0.8 to 1.0 nm. Elemental analysis was performed using an energy dispersive X-ray spectrometer (EDS) with an r-TEM detector and an 11 MPix ORIUS SC1000B (Gatan, Pleasanton, CA, USA) CCD camera. The spot/linear resolution was 0.25/0.102 nm.

### 3.6. Quantitative Phytochemical Analysis

Measurements were carried out using a double beam UV-Vis scanning spectrophotometer M550 (Spectronic CamSpec, Garforth, United Kingdom). The total phenolic content in the extracts of eucalyptus and sage leaves was determined by the Folin–Ciocalteu assay [80] and expressed as mg gallic acid equivalent (GAE) per gram of absolutely dry weight. The total content of flavonoids was determined using the described methodology [81] and expressed as mg rutin equivalent (RE) per gram of absolutely dry weight. The total content of proanthocyanidins was determined by the DMCA assay [82] and expressed as mg (-)-epicatechin equivalent (EE) per gram of absolutely dry weight. The total content of hydroxycinnamic acid derivatives was determined using the described methodology [83] and expressed as mg ChAE/g DW.

### 3.7. Evaluation of Antioxidant Activity

The ABTS^•+^ radical cation scavenging assay was applied according to the methodology described by Re et al. [63]. The DPPH^•^ free radical scavenging activity was determined using the assay proposed by Brand-Williams et al. [62]. The TFPH^•+^ radical cation scavenging assay was applied using the methodology described by Asghar and Khan [67]. The reduction activity of extracts was determined using the FRAP assay proposed by Benzie and Strain [64]. The antioxidant activity of the extracts was expressed as mmol of the Trolox equivalent (TE) per one gram of absolutely DW.

### 3.8. Antibacterial Assay

The in vitro antibacterial activity was evaluated using the Agar diffusion test against Gram-positive *Staphylococcus aureus*, *Bacillus subtilis*, *Bacillus cereus*, and *Proteus vulgaris*, and Gram-negative *Escherichia coli*, *Pseudomonas aeruginosa*, *Klebsiella pneumoniae*, and *Proteus mirabilis* bacteria strains. For this purpose, 0.5 McFarland unit density suspension (~108 CFU/mL) of bacterial strain was inoculated onto the cooled Mueller Hinton Agar (Oxoid, Basingstoke, UK), using sterile cotton swabs. Wells of 6 mm in diameter were punched in the agar and filled with 50 µL of extracts. Agar plates were incubated at 37 °C for 24 h and zones of the inhibition were measured and tabulated.

### 3.9. Statistical Analysis

All the experiments were carried out in triplicate. Means and standard deviations were calculated with STATISTICA 10 StatSoft, Inc., Tulsa, OK, USA) and Excel (Microsoft, Redmond, WA, USA) software. A one-way analysis of variance (ANOVA) with the post hoc Tukey’s HSD test was employed for statistical analysis. Differences were considered to be significant at *p* < 0.05.

## 4. Conclusions

The dispersed AgNPs were synthesized by an eco-friendly and cost-effective method using plant leaf extracts of *E. globulus* and *S. officinalis* as a reducing and capping agent. The results suggest that plant extract selection affected the morphology of nanoparticles. The size ranges of AgNPs mediated by *E. globulus* and *S. officinalis* extracts were found to be 17.5 ± 5.89 nm and 34.3 ± 7.76 nm, respectively. Flavonoids, phenolic compounds, hydroxycinnamic acid, and other phytochemicals found in plant leaf extracts and capped AgNPs are responsible for their biological activity. The biosynthesized AgNPs exerted prominent antioxidant properties and antibacterial potency against tested pathogenic bacteria strains.

## Figures and Tables

**Table 1:** Phytochemical analysis of plant extracts and biosynthesized AgNPs.

Compound Name	*E. globulus*	*S. officinalis*	*EuG*-AgNPs	*SaO*-AgNPs
The total content of proanthocyanidins, mg EE/g	0.13 ± 0.02	0.09 ± 0.00	0.09 ± 0.01	0.07 ± 0.04
The total content of hydroxycinnamic acid derivatives, mg CAE/g	1.38 ± 0.05	1.57 ± 0.02	1.24 ± 0.02	1.54 ± 0.01
The total content of phenolic compounds, mg GAE/g	0.69 ± 0.04	0.78 ± 0.00	0.58 ± 0.03	0.61 ± 0.02
The total content of flavonoids, mg RE/g	0.48 ± 0.04	0.44 ± 0.00	0.43 ± 0.01	0.41 ± 0.03

**Table 2:** Antioxidant activity of plant extracts and biosynthesized AgNPs (*p* > 0.05).

Assay	*E. globulus*	*S. officinalis*	*EuG*-AgNPs	*SaO*-AgNPs
ABTS, mmol TE/g	1.69 ± 0.07 ^c^	1.46 ± 0.04 ^d^	1.97 ± 0.01 ^b^	2.28 ± 0.04 ^a^
DPPH, mmol TE/g	0.96 ± 0.03 ^a^	0.37 ± 0.01 ^b^	0.98 ± 0.02 ^a^	0.39 ± 0.02 ^b^
TFPH, mmol TE/g	1.46 ± 0.64 ^a^	1.37 ± 0.43 ^a^	2.08 ± 0.12 ^a^	1.87 ± 0.01 ^a^
FRAP, mmol TE/g	9.23 ± 0.43 ^a^	4.23 ± 0.18 ^b^	9.11 ± 0.14 ^a^	4.02 ± 0.01 ^b^

The different superscript letters in the same line indicate statistically significant differences between the antioxidant activity of plant extracts (*p* < 0.05).

**Table 3:** Inhibition zones of the plant extracts and AgNPs against Gram-positive and Gram-negative bacteria strains (*p* > 0.05).

Bacterial Strains		Inhibition Zone ± SD, mm:			
		*E. globulus*	*S. officinalis*	*EuG-*AgNPs	*SaO-*AgNPs
Gram-positive	*S. aureus*	14.4 ± 0.01 ^d^	17.9 ± 0.20 ^c^	20.0 ± 0.10 ^b^	24.4 ± 0.05 ^a^
	*B. cereus*	13.2 ± 0.05 ^d^	15.7 ± 0.70 ^c^	20.4 ± 0.08 ^b^	24.0 ± 0.10 ^a^
	*P. vulgaris*	14.7 ± 0.10 ^c^	13.2 ± 0.55 ^d^	18.2 ± 0.09 ^b^	20.0 ± 0.22 ^a^
	*B. subtilis*	14.0 ± 0.01 ^c^	13.1 ± 0.01 ^d^	20.0 ± 0.20 ^a^	18.8 ± 0.18 ^b^
Gram-negative	*E. coli*	14.0 ± 0.02 ^c^	13.9 ± 0.01 ^d^	19.0 ± 0.01 ^b^	22.4 ± 0.03 ^a^
	*P. aeruginosa*	13.8 ± 0.01 ^c^	13.0 ± 0.10 ^d^	20.1 ± 0.03 ^b^	20.9 ± 0.27 ^a^
	*K. pneumoniae*	13.6 ± 0.04 ^c^	13.2 ± 0.25 ^d^	21.5 ± 0.01 ^b^	23.0 ± 0.14 ^a^
	*P. mirabilis*	12.5 ± 0.08 ^d^	12.8 ± 0.10 ^c^	19.7 ± 0.10 ^b^	20.7 ± 0.40 ^a^

The different superscript letters in the same line indicate statistically significant differences between Gram-positive and Gram-negative bacteria strains of plant extracts (*p* < 0.05).

## Data Availability

All data generated during this study are included in this article.
